# Similar Adiposity Improvements but Different Eating Behavior and Mental Health Responses in Men and Women: A 12-Week Exploratory Study

**DOI:** 10.3390/nu18111809

**Published:** 2026-06-04

**Authors:** María Diez-Hernández, María Fernanda Zerón-Rugerio, Isabella Parilli-Moser, Maria Izquierdo-Pulido

**Affiliations:** 1 Nutrition and Food Safety Research Institute (INSA-UB), Torribera Campus, University of Barcelona, 08921 Santa Coloma de Gramenet, Barcelona, Spain; mariadiez@ub.edu (M.D.-H.); fernanda.zeron@ub.edu (M.F.Z.-R.); iparillim@ub.edu (I.P.-M.); 2 Department of Nutrition, Food Science and Gastronomy, Food Science Torribera Campus, University of Barcelona, 08921 Santa Coloma de Gramenet, Barcelona, Spain; 3 Department of Fundamental and Clinical Nursing, Faculty of Nursing, University of Barcelona, 08907 Hospitalet de Llobregat, Barcelona, Spain

**Keywords:** sex differences, eating behaviors, mental health, body composition, diet quality, Mediterranean diet

## Abstract

**Background/Objectives:** Despite growing recognition of sex as a biological variable that may influence responses to dietary interventions, many studies still pool data from women and men. Moreover, sex-stratified changes in eating behaviors and mental health following dietary interventions remain underexplored. This study aimed to describe sex-stratified changes in adiposity, eating behaviors, and mental health outcomes in men and women following a hypocaloric Mediterranean-style diet. **Methods:** A 12-week exploratory prospective longitudinal study was conducted in nineteen women and nine men with overweight or obesity. Participants attended two clinical visits (baseline and week 12), where adiposity parameters (body mass index [BMI], body fat, waist and hip circumference), diet quality, eating behaviors, mental health parameters (well-being, perceived stress, flourishing, anxiety and depression) and biochemical parameters were assessed. Analyses were stratified by sex to evaluate changes from baseline to week 12, and repeated-measures correlations were used to explore within-individual associations between concurrent changes in outcomes. **Results:** Women and men improved significantly in adiposity and diet quality. Uncontrolled eating decreased and cognitive restraint increased in both sexes (*p* < 0.05). In men, emotional eating decreased (*p* = 0.011), and mental health improved, with higher well-being (*p* = 0.043) and flourishing (*p* = 0.027), and lower stress (*p* = 0.021), anxiety (*p* = 0.017), and depression (*p* = 0.027). Also, in men, anxiety was positively correlated with body fat percentage (*p* = 0.012) and BMI (*p* = 0.002) and inversely correlated with diet quality (*p* = 0.013). Uncontrolled eating was positively associated with BMI in men (*p* = 0.022) and women (*p* = 0.006), and cognitive restraint was positively associated with diet quality (both *p* = 0.003). **Conclusions:** Women and men achieved similar improvements in adiposity, whereas improvements in mental health variables and emotional eating were observed only in men. These preliminary findings suggest that dietary interventions may benefit from considering sex-sensitive and multidisciplinary approaches, especially psychological support and stress-management components, may be required for women. However, these observations should be confirmed in adequately powered studies.

## 1. Introduction

Obesity is recognized as an adiposity-based chronic disease and constitutes a major global public health challenge [1]. Traditionally, obesity management has focused primarily on weight loss; however, accumulating evidence indicates that, beyond changes in body weight alone, dietary interventions may confer improvements in metabolic health, eating behaviors, and well-being [2,3]. Consistently, evidence suggests that sex and gender are key modifiers of obesity risk, metabolic health, and responses to dietary and lifestyle interventions [4]. However, despite the growing recognition of sex as a key biological variable, many nutritional intervention studies continue to pool data from women and men, potentially obscuring meaningful sex-specific effects [5].

Sexual dimorphism in body composition and fat distribution is a key determinant of metabolic risk across the life span. Women generally have a higher total body fat percentage, whereas men are more prone to visceral fat accumulation and tend to present a less favorable cardiometabolic profile [4,5]. Growing evidence also suggests that sexual dimorphism likely shapes how individuals respond to dietary interventions [6,7,8]. Indeed, intervention studies based on the Mediterranean diet and low-calorie weight-loss programs consistently report that men, compared with women, usually show greater short-term reductions in body weight, fat percentage, and waist circumference [6,7]. In line with these findings, recent evidence indicates that Mediterranean and other hypocaloric interventions frequently yield greater reductions in body weight, body fat, and low-density-lipoprotein (LDL) cholesterol in men. Women, in contrast, often show smaller decreases in adiposity parameters but frequently maintain or improve high-density lipoprotein (HDL) cholesterol [8].

In this context, evidence indicates that eating behavior patterns also play a meaningful role in shaping weight-loss outcomes during dietary interventions [4,9]. Findings from an 8-week randomized controlled trial comparing a standard dietary intervention with a mindfulness-based dietary approach in adults with obesity, showed that participants assigned to the mindfulness-based program achieved greater weight loss [9]. Importantly, reductions in cognitive restraint, emotional eating, and uncontrolled eating within the mindfulness-based group were associated with lower body mass index (BMI) [9]. Despite these insights, the potential influence of sex differences on these relationships remains largely unexplored. Notably, recent findings from our research group underscored significant differences in the relationship between obesity and eating behaviors. Our results showed that, in women but not in men, emotional and uncontrolled eating emerged as key pathways linking poorer well-being, with greater adiposity [10]. These findings suggest that psychological and behavioral mechanisms underlying obesity may differ between sexes.

Along these lines, obesity has consistently been associated with poor mental health outcomes [10,11]. For instance, women are two to three times more likely than men to experience mental health problems, including anxiety and mood disorders [12]. Therefore, given that food intake can serve as a coping mechanism for negative emotions, it is plausible that sex differences in psychological vulnerability could translate into differential eating behavior patterns. Additionally, biological mechanisms, including differences in stress physiology and sex hormones, as well as psychosocial factors such as body image concerns and weight stigma, may further contribute to these disparities [12,13]. Together, these factors suggest that sex differences in psychological and biological vulnerability may contribute to different eating-behavior profiles, with potential implications for both obesity risk and responsiveness to dietary interventions.

Considering the aforementioned, this exploratory study aimed to describe sex-specific changes in adiposity, eating behaviors, and mental health outcomes in adults with overweight or obesity following a hypocaloric Mediterranean-style diet for 12 weeks. In addition, we explored whether within-individual changes in adiposity parameters and diet quality were associated with concurrent changes in eating behaviors and mental health outcomes when analyses were stratified by sex.

## 2. Materials and Methods

### 2.1. Subjects

This exploratory prospective longitudinal study included adults with overweight or obesity who had previously taken part in a cross-sectional study [10]. After completion of that study, eligible individuals were invited to join a 12-week weight loss program conducted at the Hospital de la Santa Creu i Sant Pau in Barcelona, Spain. Participants were eligible if they were women or men aged 25–50 years and had a BMI of 25–35 kg/m^2^. These age and BMI ranges were selected to obtain a relatively homogeneous population, minimizing the influence of major physiological and lifestyle transitions while focusing on metabolically stable individuals for whom dietary and lifestyle interventions are an appropriate first-line treatment [14,15,16,17]. In addition, individuals were eligible to participate if they were metabolically healthy, defined as the absence of diagnosed metabolic diseases together with normal biochemical parameters at screening, and agreed to participate. Exclusion criteria included food allergies or intolerances, obesity-related comorbidities (such as type 2 diabetes mellitus, hypertension, or dyslipidemia), diagnosed gastrointestinal diseases, current smoking, shift work, diagnosed eating disorders, use of medication including antidepressant or mood-improving medication, weight loss treatment within the previous three months, or a history of bariatric surgery or other bariatric procedures. Additionally, women who were pregnant, breastfeeding, in menopause or post-menopause were excluded. As shown in Figure S1, 123 individuals were invited to participate, of whom 29 fulfilled the eligibility criteria and were enrolled. During follow-up, one participant was lost, resulting in a final analytical sample of 28 participants.

### 2.2. Study Protocol

**Figure 1:**
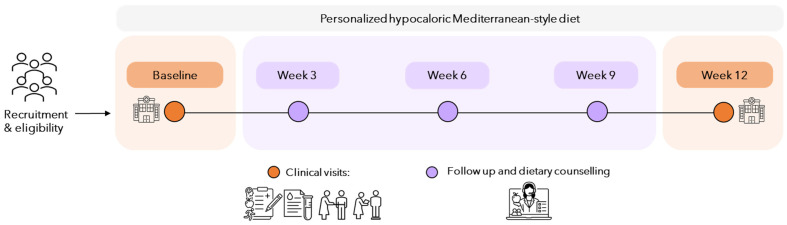
Study design.

All participants attended two on-site clinical visits: baseline (week 0) and end of the study (week 12) (Figure 1). At baseline, adherence to the Mediterranean diet was assessed using the 17-item Mediterranean Diet Adherence Screener (MEDAS-17) to characterize baseline diet quality and to identify which Mediterranean diet components required greater reinforcement in each participant. Based on this evaluation, participants received an individualized hypocaloric Mediterranean-style dietary plan, consisting of written dietary recommendations, example menus, portion-size guidance, and nutritional counseling aimed at improving adherence to Mediterranean diet principles. Individual energy requirements were estimated from resting metabolic rate using the Mifflin-St. Jeor equation [18] and adjusted according to physical activity level. A moderate energy deficit of approximately 500 kcal/day was then applied to establish individualized energy requirements, which ranged from 1200 to 1800 kcal/day. The prescribed diet followed Mediterranean diet principles and provided a macronutrient distribution of 30–35% fat, 45–50% carbohydrate, and 20–25% protein [17].

In addition, to promote adherence, participants attended three online follow-up sessions (weeks 3, 6, and 9) led by a nutritionist, who provided continuous dietary counseling and adapted dietary recommendations when necessary. Throughout the study, participants were not informed of their body weight or anthropometric measurements, including body fat percentage, muscle mass, or waist and hip circumference. Participants were also instructed not to weight themselves at home, and this recommendation was reinforced at each clinical visit and follow-up contact. This approach was implemented to minimize weight-centered focus and to encourage participants to pay greater attention to subjective indicators of health and well-being.

### 2.3. Ethical Statement

The study was conducted according to the Declaration of Helsinki and received approval from the Ethics Committee of Drugs Research at the Hospital de la Santa Creu i Sant Pau (IIBSP-SOB-2022-109). All participants provided written informed consent before being included in the study.

### 2.4. Study Outcomes

The primary outcome of the study was the change in adiposity-related parameters from baseline to week 12, including body weight, BMI, body fat percentage, waist circumference, hip circumference, and waist-to-hip ratio. Secondary outcomes included changes in eating behaviors, mental health-related parameters, Mediterranean diet adherence, physical activity, and biochemical parameters.

#### 2.4.1. Adiposity Parameters

Body weight and body composition were assessed at baseline and at the end of the study using a bioelectrical impedance analysis device (InBody 120; Biospace, Seoul, Republic of Korea) [19]. Measurements were performed with participants wearing light clothing and without shoes. To prevent participants from viewing their results, the device display was covered during all assessments. Height was measured to the nearest 0.1 cm using a fixed stadiometer (Seca 217; Seca, Hamburg, Germany) [20]. BMI was calculated as body weight in kilograms divided by height in meters squared [13].

Waist and hip circumferences were measured to the nearest 0.1 cm with a flexible, non-elastic anthropometric tape (Cescorf, Porto Alegre, Brazil) calibrated in centimeters. Waist circumference was assessed at the midpoint between the lower margin of the rib and the iliac crest, with the participant standing and after a gentle expiration. Hip circumference was measured at the level of the greater trochanter, at the widest portion of the buttocks. Waist-to-hip ratio was obtained by dividing waist circumference (cm) by hip circumference (cm). Abdominal obesity was defined according to World Health Organization criteria as waist-to-hip ratio ≥0.85 in women and ≥0.90 in men [21].

The percentages of weight loss, fat loss, muscle mass loss, BMI loss, waist loss and hip loss were calculated in each case using the following formula: [(baseline values − week 12 values)/baseline values] × 100.

#### 2.4.2. Eating Behaviors

Eating behaviors were evaluated during clinical visits (baseline and week 12), using the Spanish version of the Three Factor Eating Questionnaire (TFEQ-R21) [22]. This questionnaire assesses three dimensions of eating behavior:

- Emotional eating was assessed through six items and is defined as the need to eat when an individual is unable to effectively cope with negative emotional situations.
- Cognitive restraint was assessed through six items and refers to the conscious effort made by the individual to control food intake in order to maintain or reduce body weight.
- Uncontrolled eating was assessed through nine items and is defined as the propensity to overeat triggered by a perceived loss of control over food intake.

Scores for each dimension were computed separately as the mean of all items, with higher scores indicating higher emotional eating, cognitive restraint, and/or uncontrolled eating [22,23]. In the present sample, the TFEQ-R21 showed good internal consistency across all subscales, with Cronbach’s alpha values of 0.859 for emotional eating, 0.896 for uncontrolled eating, and 0.806 for cognitive restraint.

#### 2.4.3. Parameters Related to Mental Health

During clinical visits, the following parameters were assessed using validated questionnaires for the Spanish population:

- Well-being was measured with the Spanish version of the WHO-5 questionnaire [24], a 5-item self-administered scale used to evaluate participants’ well-being during the preceding two weeks. Scores range from 0 to 100, with higher values indicating better well-being [25,26].
- Perceived stress was evaluated through the 10-item Perceived Stress Scale (PSS-10) [27]. This self-reported instrument evaluates the extent to which individuals perceive situations in their lives over the past month as unpredictable, uncontrollable, and/or stressful. Scores range from 0 to 40, where higher scores indicate greater perceived stress [27].
- Anxiety and depression were assessed with the Hospital Anxiety and Depression Scale (HADS) [28], a 14-item self-report scale consisting of a 7-item anxiety subscale (HADS-A) and a 7-item depression subscale (HADS-D). Scores range from 0 to 21 for each subscale, where higher scores indicate greater anxiety and/or depression symptoms [28].
- Flourishing was evaluated through the Flourishing Scale [29], an 8-item self-reported instrument that evaluates key aspects of human functioning, including positive relationships, feelings of competence, and having meaning and purpose in life. Scores range from 8 to 56, where higher scores indicate higher flourishing [29].

Most mental health-related parameters showed good internal consistency, with Cronbach’s alpha coefficients of 0.893 for WHO-5, 0.851 for PSS-10, 0.812 for HADS-D, and 0.787 for flourishing. The HADS-A exhibited moderate internal consistency, with a Cronbach’s alpha coefficient of 0.713.

#### 2.4.4. Lifestyle Variables

Adherence to the Mediterranean diet was assessed during clinic visits using the MEDAS-17, which has been validated in the Spanish population [30]. Scores range from 0 to 17, with higher scores indicating greater adherence to the Mediterranean diet.

Physical activity levels were measured in Metabolic Equivalents of Task (METs) using the short version of the International Physical Activity Questionnaire (IPAQ), also validated in the Spanish population [31]. Higher scores reflect a greater intensity of physical activity.

#### 2.4.5. Biochemical Parameters

At the two clinical visits, participants were instructed to attend the Hospital de la Santa Creu i Sant Pau for venous blood sampling after a 12-h overnight fast. Blood draws were performed by trained nursing staff at the hospital. Samples were then processed and analyzed in the hospital’s chemical laboratory according to standard laboratory procedures. Biochemical parameters related to glycemic control and lipid profile were assessed, including glucose, glycated hemoglobin (HbA1c), triglycerides, total cholesterol, HDL cholesterol, and LDL cholesterol. Additionally, as a marker of insulin resistance, the triglyceride-glucose (TyG) index was calculated using the following formula: TyG = ln (triglycerides [mg/dL] × glucose [mg/dL]/2) [32]. Higher TyG index values indicate greater insulin resistance or a less favorable cardiometabolic profile.

#### 2.4.6. Sociodemographic Variables

Participants self-reported their sex and date of birth (to calculate age) through standardized questions. Educational level and employment status were assessed using the following questions: ‘What is your education level?’ (Response options: “primary studies” or “more than primary studies”) and ‘What is your current employment status?’ (Response options: “employed”, “student” or “unemployed”).

### 2.5. Statistical Analyses

All analyses were conducted separately and stratified by sex (women and men). The distribution of continuous variables was assessed using the Shapiro–Wilk test. Variables are described by means and standard deviations. Changes in main and secondary outcomes from baseline to week 12 were assessed using paired *t*-tests for normally distributed variables or Wilcoxon signed-rank tests for non-normally distributed variables. Differences in the percentage of weight, body fat, and muscle mass loss between men and women were tested using independent-samples *t*-tests. These analyses were conducted in SPSS version 27 (IBM, Chicago, IL, USA).

Then, repeated-measures correlation analyses were conducted to examine within-individual associations between concurrent changes in adiposity parameters and diet quality with eating behaviors and parameters related to mental health. This statistical approach enables the assessment of within-individual associations for paired measures obtained on two or more time points across multiple individuals. At the same time, it accounts for the non-independence of repeated observations by applying analysis of covariance to adjust for inter-individual variability in study outcomes. These analyses were conducted using the ‘rmcorr’ [33] package in R Software (RStudio version 2025.05.0+496, R Foundation, Vienna, Austria).

As a complementary exploratory approach, linear mixed-effects models were used to examine the associations of adiposity parameters and diet quality with eating behaviors and parameters related to mental health. Continuous variables were mean-centered to facilitate the interpretation of main effects in the presence of interaction terms. Fixed effects included the main predictors (adiposity parameters, diet quality, eating behaviors and parameters related to mental health), sex, time (baseline vs. 12 weeks), and their interaction terms with sex. A random intercept for participant was included to account for within-subject variability, whereas time was included as a fixed effect to account for overall changes between baseline and 12-week follow-up. Interactions between predictors and sex were tested to determine whether associations differed between men and women. Results are presented as regression coefficients (β), 95% confidence intervals, and *p*-values. These analyses were conducted using the ‘lmer’ package [34] in R Software (RStudio version 2025.05.0+496, R Foundation, Vienna, Austria). For all analyses, statistical significance was inferred when *p*-value was <0.05.

Because this was a secondary exploratory analysis based on available data, no a priori sample size calculation was performed specifically for the present sex-stratified objectives. To contextualize statistical power, a sensitivity power analysis was conducted. With the available sample size of 19 women and 9 men, the study had 80% power to detect only large between-sex differences in change scores, corresponding to a minimum detectable effect size of Cohen’s d = 1.18, assuming a two-sided alpha level of 0.05.

## 3. Results

A total of twenty-eight participants with overweight or obesity, nineteen women (BMI: 32.8 ± 4.8 kg/m^2^) and nine men (BMI: 33.4 ± 4.2 kg/m^2^) were included in this exploratory prospective longitudinal study. Baseline sociodemographic characteristics were similar between sexes, with no significant differences in age (men: 36.6 ± 7.6 years and women: 36.9 ± 7.8 years, *p* = 0.921), education levels (university degree: men 84.2% and women 88.9%, *p* = 0.741) or employment status (100% employed in both groups).

### 3.1. Within-Group Changes in Outcome Variables Throughout the Study

**Figure 2:**
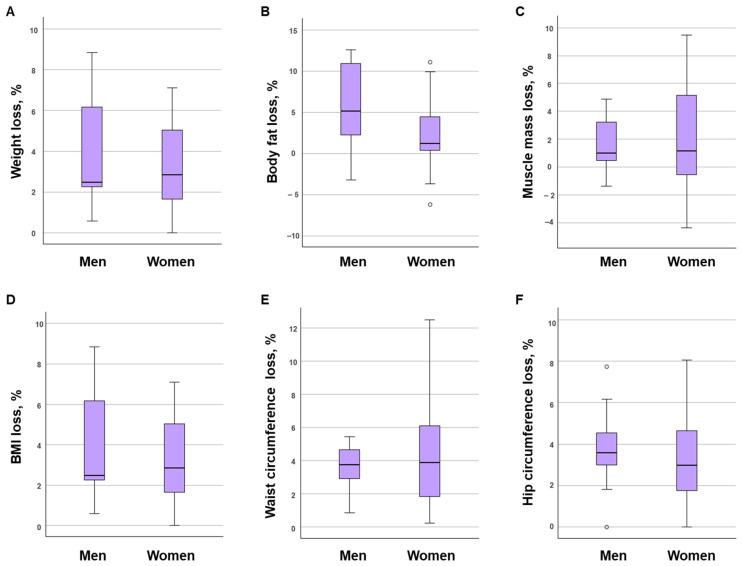
Boxplots of the changes in adiposity parameters between women and men from baseline to week 12. Figure Panels show the percentage of weight loss (**A**), body fat loss (**B**), muscle mass loss (**C**), BMI loss (**D**), waist circumference loss (**E**), and hip circumference loss (**F**). The central line indicates the median, the box boundaries represent the interquartile range, the whiskers indicate the range of the data, and points outside the whiskers are outliers. Statistical comparisons between groups were performed using *t*-test, with *p* < 0.05 considered statistically significant.

As shown in Table 1, both women and men showed significant reductions in BMI (women: *p* < 0.001; men: *p* = 0.003), waist circumference (*p* < 0.001 in both sexes), and hip circumference (women: *p* < 0.001; men: *p* = 0.001) from baseline to week 12. In addition, a statistically significant decrease in body fat percentage was observed in men (*p* = 0.044), whereas this change in women was close to statistical significance (*p* = 0.053). Despite these differences, Figure 2 suggests similar percentages of weight, body fat, muscle mass, BMI, waist and hip loss in women and men at week 12. 

Regarding lifestyle-related variables, diet quality improved significantly from baseline to week 12 in both women and men (*p* < 0.001, for both), while physical activity remained stable (Table 1). Our results also showed that emotional eating remained unchanged in women, whereas a statistically significant decrease was observed in men (*p* = 0.011). In addition, both women and men showed a significant reduction in uncontrolled eating scores (*p* = 0.025 and *p* = 0.023, respectively) and a significant increase in cognitive restraint scores (*p* = 0.034 and *p* = 0.036, respectively) (Table 1).

Concerning mental health outcomes, in men, we observed that stress, anxiety, and depression levels were lower at week 12 (*p* = 0.021, *p* = 0.017 and *p* = 0.027, respectively). Conversely, at week 12, men exhibited higher well-being and flourishing scores (*p* = 0.043 and *p* = 0.027, respectively). In contrast, no significant changes were observed in women, although a trend toward lower anxiety was noted (*p* = 0.066) (Table 1).

Concerning biochemical parameters (Table 1), both women and men showed significant reductions in HbA1c (*p* = 0.015 and *p* = 0.035, respectively) from baseline to week 12. Meanwhile, in women only, HDL cholesterol (*p* = 0.033) decreased after 12 weeks.

### 3.2. Repeated Measures Correlations Between Adiposity Parameters, Diet Quality, Eating Behaviors and Parameters Related to Mental Health

**Figure 3:**
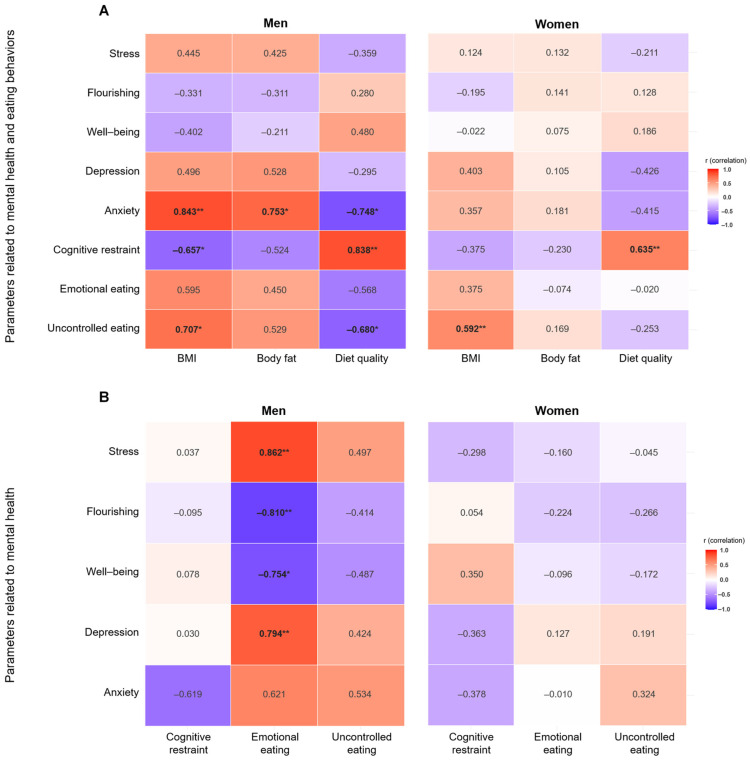
Heatmap of repeated-measures correlation coefficients stratified by sex. Figure (**A**) shows the correlation between adiposity parameters (BMI, body fat) and diet quality with eating behaviors and parameters related to mental health. Figure (**B**) shows correlations between eating behaviors and parameters related to mental health. The color gradient indicates the strength and direction of the correlations, ranging from deep red, indicating strong positive correlations (r values near 1), to deep blue, indicating strong negative correlations (r values near −1). The numerical values within each cell represent the repeated-measures correlation coefficients. * *p* < 0.05, ** *p* < 0.010.

As shown in Figure 3A, concurrent changes in adiposity parameters and diet quality were significantly associated with mental health variables in men. Anxiety was positively correlated with body fat percentage (r = 0.753; *p* = 0.012) and BMI (r = 0.843; *p* = 0.002) and inversely correlated with diet quality (r = −0.748; *p* = 0.013). In contrast, no significant correlations were observed in women.

Regarding eating behaviors (Figure 3A), concurrent changes in cognitive restraint were positively correlated with diet quality in both sexes (men: r = 0.838; *p* = 0.003 and women: r = 0.635; *p* = 0.003). Likewise, uncontrolled eating scores were positively correlated with higher BMI in both men (r = 0.707; *p* = 0.022) and women (r = 0.592; *p* = 0.006). Additionally, in men, cognitive restraint was inversely correlated with BMI (r = −0.657; *p* = 0.039) and uncontrolled eating was inversely correlated with diet quality (r = −0.680; *p* = 0.031).

Notably, concurrent changes in emotional eating were significantly associated with mental health parameters in men (Figure 3B). Higher emotional eating scores were positively correlated with stress (r = 0.862; *p* = 0.001) and depression (r = 0.794; *p* = 0.006) and inversely correlated with well-being (r = −0.754; *p* = 0.012) and flourishing (r = −0.810; *p* = 0.004). In contrast, no significant correlations between emotional eating and mental health variables were detected in women.

### 3.3. Complementary Exploratory Analyses: Interaction Effects of Sex and Study Outcomes

As part of complementary exploratory analyses, sex interactions with outcome variables were examined. As shown in Table S1, significant interactions with sex were observed for both anxiety and uncontrolled eating in relation to diet quality. Specifically, a significant anxiety × sex interaction was identified (β = 0.78, 95% CI: 0.22 to 1.34, *p* = 0.007), indicating that higher anxiety was associated with lower diet quality in men (β = −0.71), whereas this association was attenuated. Similarly, uncontrolled eating showed a significant interaction with sex (β = 3.10, 95% CI: 1.13 to 5.07, *p* = 0.003). In men, higher uncontrolled eating was associated with lower adherence to the Mediterranean diet (β = −2.45), while in women this association was attenuated.

For BMI, a significant anxiety × sex interaction was also observed (β = −0.32, 95% CI: −0.62 to −0.01, *p* = 0.043). In men, higher anxiety was associated with higher BMI (β = 0.34), whereas this association was negligible in women.

Although no other significant interactions were observed for the remaining outcomes, these exploratory findings suggest that the associations between psychological factors and both diet quality and BMI may differ by sex.

## 4. Discussion

The main finding of this exploratory longitudinal study is that, although women and men achieved similar improvements in adiposity-related outcomes and diet quality, statistically significant improvements in emotional eating and several mental health-related parameters and emotional eating were detected only in men. Notably, from baseline to week 12, men showed lower emotional eating, stress, anxiety, and depressive symptoms, along with higher well-being and flourishing scores. In addition, repeated-measures correlations suggested that the decrease in emotional eating scores in men was accompanied by lower stress and depression, while well-being and flourishing scores increased. In contrast, women showed no significant correlations between these parameters, suggesting that the psychological response pattern associated with dietary modification may not be the same in women and men. These results were supported by mixed-effects models, which showed that higher anxiety and uncontrolled eating were associated with poorer diet quality in men, whereas these relationships were weaker or absent in women. Additionally, anxiety was positively associated with BMI in men but not in women, indicating sex-specific patterns in the link between psychological factors, diet quality, and adiposity.

Overall, the results observed in men are consistent with previous studies indicating that improvements in diet quality may positively influence mental health by reducing depressive symptoms, stress, and anxiety [35], and by enhancing well-being [2]. However, a recent systematic review and meta-analysis reported improvements in depressive symptoms, stress, and anxiety in women only after lifestyle interventions, including dietary changes [36]. Our results are also consistent with current evidence, linking emotional eating to negative psychological states, and emotional dysregulation [37]. Noteworthy, Mento et al. [38] suggested that, in women with obesity, negative emotions and maladaptive eating-related traits were more tightly interconnected than in men. Specifically, they found that women exhibited a substantially higher number of correlations between negative mood states and eating-disorder-related dimensions, whereas these associations were fewer and more sporadic in men, which is consistent with previous findings of our research group [10]. Similarly, in a large multinational analysis from the MEDIET4ALL project, women reported significantly higher depression, anxiety, and stress, as well as greater perceived psycho-social, physical, and nutritional support needs, whereas men more often reported no need for support [39]. Although these findings do not directly explain the present results, they suggest that women with overweight or obesity may present more complex psychosocial needs during weight-loss interventions. From a practical perspective, this supports the need to consider multidisciplinary approaches that combine nutritional counseling with psychological support, stress-management strategies, and specific tools to address emotional eating, particularly in women.

Along these lines, the lack of significant improvements in emotional eating in women may reflect a combination of biological and psychosocial factors. Women are more prone to emotional eating than men, possibly due to differences in the physiological response to stress [40] and the influence of gonadal hormones [12]. Women often show lower hypothalamic–pituitary–adrenal (HPA) axis and autonomic stress responses than men. Given that acute stress responses may suppress appetite via HPA activation, a relatively attenuated physiological stress response could contribute to greater reliance on food as an emotion regulation strategy [40,41]. Furthermore, fluctuations in the estradiol and progesterone across the menstrual cycle, particularly during the mid-luteal phase, have been associated with increased vulnerability to emotional and/or binge eating, while testosterone appears to have the opposite effect [12]. Beyond biological mechanisms, psychosocial factors may also play a key role. Women are generally more vulnerable to body image-related stressors and weight stigma, which may attenuate psychological improvements during weight-loss programs even when dietary adherence is achieved [42]. This interpretation is compatible with our findings.

Among other relevant findings, repeated-measures correlation analyses suggested that, in men, improvements in diet quality and reductions in adiposity were associated with lower anxiety levels. Consistently, significant sex-by-anxiety interactions suggested that higher anxiety was associated with higher BMI and poorer diet quality in men. In line with our findings, previous studies have shown that greater adherence to the Mediterranean diet can positively influence mood and anxiety [43,44]. Two recent cross-sectional studies have reported significant associations between the Mediterranean diet and a lower risk of anxiety and depression in Italian adults with obesity [43] and in Iranian adults [44]; however, these studies evaluated the overall sample without examine sex-specific differences. These associations may be explained by the fact that the Mediterranean diet is rich in bioactive compounds and nutrients involved in the synthesis of serotonin, dopamine, and gamma-aminobutyric acid; neurotransmitters closely related to mood regulation, anxiety, and well-being [45].

The positive relationship between concurrent changes in adiposity and anxiety is also supported by a systematic review and meta-analysis showing that individuals with overweight or obesity report higher anxiety levels than those without obesity [46]. Accordingly, symptoms of anxiety may increase appetite and the preference for palatable “comfort foods” as a coping strategy, thereby reinforcing a cycle that favors weight gain [47]. In our study, we also observed sex-specific patterns, such as higher anxiety and uncontrolled eating were associated with poorer diet quality in men, whereas this relationship was attenuated in women. Along these lines, evidence suggests that cultural and social factors, such as body stigma, appearance norms, self-image, and socioeconomic status, may modulate the association between anxiety and obesity [46]. Women, for example, tend to report greater body dissatisfaction and experience more severe weight-related stigma and discrimination than men [46]. Beyond anxiety, evidence also supports a link between adiposity and depressive symptoms. A meta-analysis including 17 studies totaling 204,507 participants, found a significant positive association between obesity and depression in the general population, and this association was stronger in women [48].

Regarding eating behaviors, both sexes showed less uncontrolled eating and higher cognitive restraint at week 12 compared with baseline. These results are consistent with previous intervention studies showing that dietary weight loss interventions improve eating behaviors in individuals with overweight or obesity [9,49]. Such changes may reflect greater self-regulation and improved adherence to dietary goals, which have been identified as predictors of successful weight management [50]. In this context, the positive correlation between cognitive restraint and diet quality in both sexes may reflect the adoption of healthier habits during a structured dietary program rather than maladaptive restriction. In men, concurrent changes in cognitive restraint were additionally associated with lower BMI. It is possible that cognitive restraint may play a role in weight regulation in men during structured dietary counseling, although given the exploratory nature of this study, these findings warrant further investigation. Moreover, increased cognitive restraint should be interpreted with caution, as restraint theory suggests that chronic dietary restriction may alternate with overeating episodes, potentially leading to a vicious circle of weight regain over time [22].

Regarding adiposity parameters, both men and women showed significant reductions in BMI and central adiposity, as reflected by decreases in waist and hip circumference. Interestingly, men, but not women, showed a decrease in body fat percentage after 12 weeks of a hypocaloric Mediterranean-style diet. These results are consistent with a systematic review that concludes that dietary interventions based on the Mediterranean diet reduce central obesity and lower the risk of obesity-related chronic diseases [51].

Biochemical changes were modest overall. The significant reduction in HbA1c observed in both women and men is in line with evidence from a meta-analysis indicating that greater adherence to the Mediterranean diet improves glycemic control [52]. By contrast, HDL cholesterol decreased significantly in women only in this sample. However, these results should be interpreted with caution considering that the effect of weight loss on HDL cholesterol is heterogeneous and may depend on factors such as the magnitude of energy restriction, the dynamics of weight loss, and dietary composition [53].

Taken together, these findings are in line with previous evidence suggesting the potential importance of adopting a sex-sensitive approach in nutritional interventions for obesity. Current obesity management guidelines emphasize that obesity is a chronic, complex, and multifactorial disease that requires an individualized and multidisciplinary approach [17]. In line with this perspective, eating behaviors and mental health-related parameters may represent relevant dimensions to consider when designing personalized dietary interventions for obesity management. While men in this study appeared to experience both physical and psychological benefits from the interventions, futures studies in women should examine whether more integrated approaches combining dietary modification with psychological support, stress-management strategies, or targeted support for emotional eating may improve intervention efficacy.

However, the interpretation of these results should consider several methodological limitations. First, this study was conducted as a secondary exploratory analysis based on available data from participants who completed a 12-week hypocaloric Mediterranean-style diet. Therefore, the single-arm design and the absence of a control group preclude causal inference, and the observed changes may have been influenced by factors other than the dietary intervention itself. Second, the relatively small sample size, particularly the limited number of men, should be considered when interpreting the sex-specific findings. The sensitivity power analysis indicated that the available sample size was sufficient to detect only large between-sex differences; therefore, smaller but potentially clinically relevant effects may have remained undetected. Third, participants were recruited from a selected group of volunteers previously involved in a related study, which may have introduced selection bias and limited the generalizability of the findings. Moreover, although participants were repeatedly instructed not to weigh themselves at home and to maintain their usual lifestyle habits, self-weighing and other lifestyle behaviors outside the study setting could not be objectively monitored. Fourth, menstrual cycle phase was not recorded or controlled in women at the time of questionnaire administration; consequently, hormonal fluctuations across the menstrual cycle may have contributed to variability in emotional eating and psychological outcomes. Future studies should consider recording menstrual cycle phase and, when feasible, standardizing assessments at comparable phases of the cycle. Fifth, while the impact of sex has been examined in prior studies, the topic is not entirely novel, yet, the present work adds to this body of evidence by offering an integrated evaluation of adiposity outcomes together with eating behaviors and mental health–related parameters during a structured dietary weight-loss intervention. However, based on these limitations, the present findings should be interpreted as preliminary and hypothesis-generating, and future controlled intervention studies are warranted to confirm these exploratory results.

Despite these limitations, the study also has notable strengths, including its prospective longitudinal design, the use of validated instruments to assess eating behaviors and mental health, and the incorporation of sex-stratified within-person exploratory analyses.

## 5. Conclusions

In this exploratory longitudinal study, a 12-week hypocaloric Mediterranean-style diet was associated with improvements in adiposity parameters, diet quality, and uncontrolled eating in men and women with overweight and obesity. However, statistically significant improvements in stress, anxiety, depression, well-being, flourishing, and emotional eating were detected only in men. Within-individual analyses further suggested that, in men, improvements in diet quality and reductions in adiposity and emotional eating were associated with better mental health, whereas comparable physical improvements in women were not accompanied by statistically significant mental health parameters. Yet, larger studies with longer follow-up periods are warranted to confirm these results and to clarify whether a sex-sensitive approach in dietary weight-loss interventions, with added psychological support and stress-management components, may be required for women.

## Figures and Tables

**Table 1:** Baseline characteristics and changes from baseline to week 12 in adiposity parameters, lifestyle variable, eating behaviors and parameters related to mental health, stratified by sex.

	Women (n = 19)		*p*-Value	Men (n = 9)		*p*-Value
	Baseline Mean (SD)	Week 12 Mean (SD)		Baseline Mean (SD)	Week 12 Mean (SD)	
**Adiposity parameters**						
BMI, kg/m^2^	32.8 (4.8)	31.7 (4.5)	**<0.001**	33.4 (4.2)	32.0 (4.1)	**0.003**
Body weight, kg	88.0 (13.1)	85.2 (12.6)	**<0.001**	108.9 (14.7)	104.1 (13.7)	**0.003**
Body fat ^†^, %	44.4 (6.0)	43.5 (7.0)	0.053	35.4 (7.1)	33.5 (7.8)	**0.044**
Muscle mass ^†^, kg	26.8 (3.0)	26.3 (3.3)	0.058	39.8 (4.9)	39.1 (4.7)	0.050
Waist circumference, cm	90.6 (6.7)	86.9 (6.6)	**<0.001**	109.0 (9.3)	105.0 (9.2)	**<0.001**
Hip circumference, cm	118.3 (9.9)	114.4 (9.1)	**<0.001**	119.3 (8.6)	114.8 (9.0)	**0.001**
Waist-to-hip ratio, a.u.	0.8 (0.1)	0.8 (0.1)	0.267	0.9 (0.1)	0.9 (0.1)	0.927
**Lifestyle variables**						
Diet quality, score	7.8 (2.3)	11.1 (1.6)	**<0.001**	5.0 (2.9)	10.1 (2.6)	**<0.001**
Physical activity ^†^, METs	1990.9 (1323.7)	2252.1 (1319.4)	0.248	1549.3 (1239.2)	1765.1 (1359.4)	0.594
**Eating behaviors**						
Emotional eating ^†^, score	2.3 (0.7)	2.2 (0.8)	0.554	2.2 (0.5)	1.6 (0.4)	**0.011**
Uncontrolled eating, score	2.1 (0.6)	1.9 (0.5)	**0.025**	2.5 (0.7)	1.9 (0.5)	**0.023**
Cognitive restraint, score	2.4 (0.6)	2.6 (0.6)	**0.034**	2.1 (0.4)	2.5 (0.5)	**0.036**
**Parameters related to mental health**						
Stress ^†^, score	16.1 (6.0)	15.0 (6.5)	0.326	16.8 (7.4)	11.2 (4.7)	**0.021**
Anxiety ^†^, score	7.4 (3.2)	6.4 (3.7)	0.066	7.7 (2.4)	6.0 (1.5)	**0.017**
Depression ^†^, score	4.6 (3.8)	3.4 (2.7)	0.241	4.2 (1.8)	2.0 (1.2)	**0.027**
Well-being, score	56.6 (23.0)	55.2 (20.2)	0.705	54.7 (10.6)	68.0 (13.1)	**0.043**
Flourishing ^†^, score	49.3 (5.0)	50.1 (5.7)	0.247	47.7 (4.6)	51.2 (3.9)	**0.027**
**Biochemical parameters**						
Glucose, mg/100 mL	80.9 (6.5)	82.4 (7.3)	0.164	87.2 (9.4)	83.8 (4.4)	0.306
HbA1c ^†^, %	5.3 (0.2)	5.2 (0.2)	**0.015**	5.3 (0.3)	5.1 (0.2)	**0.035**
Triglycerides ^†^, mg/100 mL	83.5 (39.5)	67.8 (19.5)	0.073	129.8 (56.0)	111.4 (34.9)	0.260
TyG index, a.u.	8.0 (0.4)	7.9 (0.3)	0.084	8.5 (0.5)	8.4 (0.3)	0.228
Total cholesterol, mg/100 mL	187.7 (17.9)	183.7 (20.2)	0.204	195.5 (36.0)	187.0 (38.1)	0.158
HDL cholesterol, mg/100 mL	60.9 (7.2)	57.6 (8.9)	**0.033**	37.6 (8.3)	38.9 (7.3)	0.300
LDL cholesterol, mg/100 mL	110.0 (16.9)	112.4 (20.0)	0.328	131.7 (29.5)	125.7 (34.4)	0.343

BMI, Body mass index; METs, Metabolic Equivalents of Task; HbA1c, Glycated hemoglobin; TyG, triglyceride-glucose; HDL, high density lipoprotein; LDL, low density lipoprotein; SD, Standard Deviation. Data are expressed as mean (standard deviation). ^†^ Indicates variables with a non-normal distribution. Comparisons between baseline and week 12 were performed separately for women and men using paired *t*-tests for normally distributed variables or Wilcoxon signed-rank tests for non-normally distributed variables. Significant *p*-values (*p* < 0.05) are shown in bold.

## Data Availability

Upon request, data described in the manuscript will be made available.

## Supplementary Materials

The following supporting information can be downloaded at: https://www.mdpi.com/article/10.3390/nu18111809/s1. Figure S1. Flow diagram of participants included in the study. Table S1. Mixed–Effects Regression Models.
