# Supplementary material for: Similar Adiposity Improvements but Different Eating Behavior and Mental Health Responses in Men and Women: A 12-Week Exploratory Study
Source: Nutrients. 2026 Jun 4;18(11):1809. doi: 10.3390/nu18111809 (PMC13258816; doi:10.3390/nu18111809)
Supplement: Supplementary file 1 [file nutrients-18-01809-s001.zip › nutrients-4307541-supplementary.pdf]

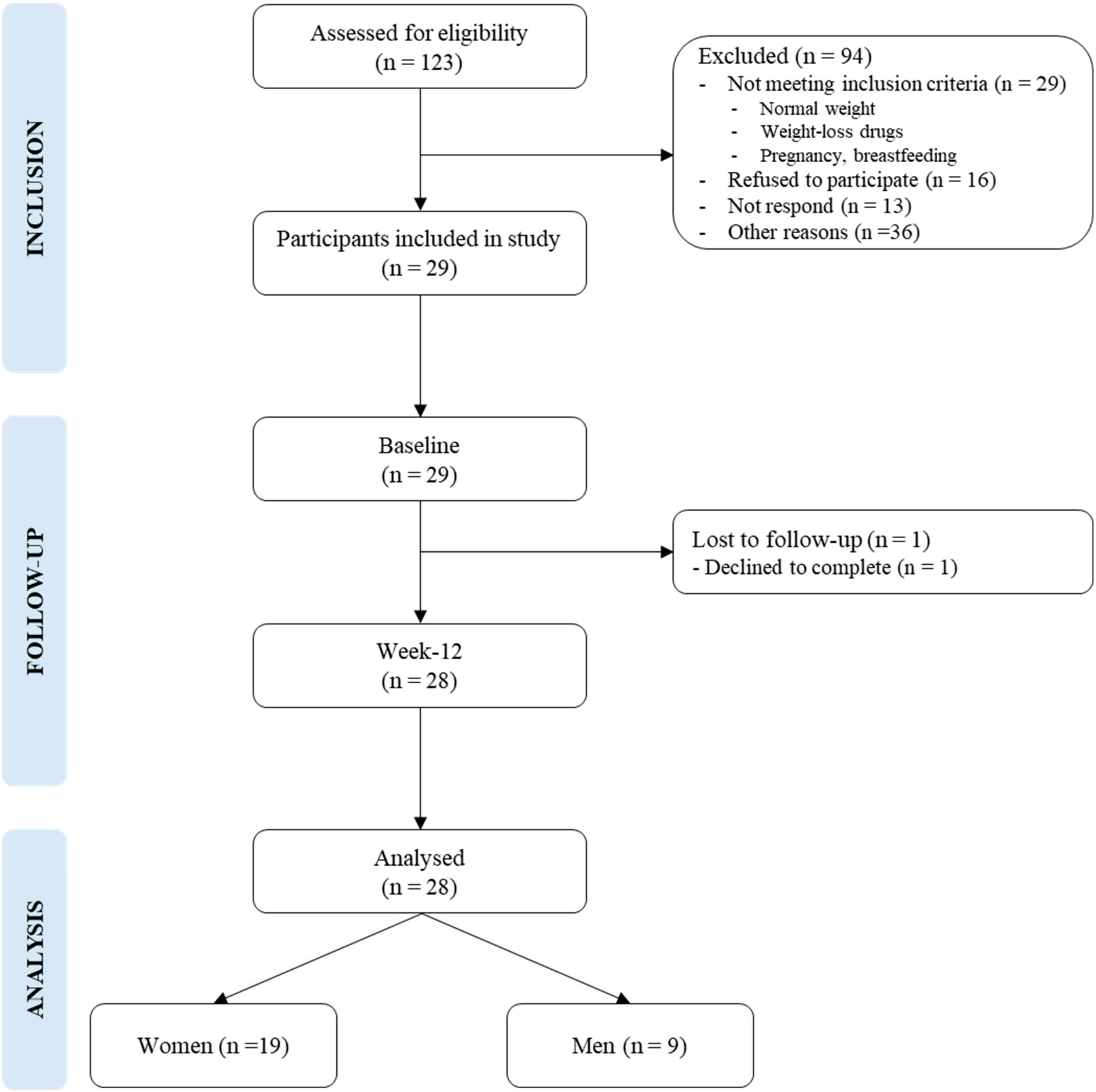

Figure S1. Flow diagram of participants included in the study.

Table S1. Mixed–Effects Regression Models.

		BMI, kg/m²	Fat mass, %	Diet quality, score	Emotional eating, score	Uncontrolled eating, score	Cognitive restraint, score
Anxiety	Anxiety (centered), score	0.34 [0.07; 0.62]*	0.64 [0.02; 1.27]*	–0.71 [–1.23; –0.19]**	0.01 [–0.15; 0.17]	0.13 [0.01; 0.25]*	–0.06 [–0.18; 0.07]
	Sex (women)[a]	–0.41 [–4.07; 3.26]	9.48 [3.85; 15.11]**	1.92 [0.49; 3.35]*	0.36 [–0.10; 0.82]	–0.15 [–0.52; 0.23]	0.21 [–0.21; 0.63]
	Time (12 weeks)[b]	–0.99 [–1.37, –0.61]***	–0.79 [–1.66; 0.07]*	3.55 [2.50; 4.60]***	–0.23 [–0.53; 0.08]	–0.23 [–0.44; –0.01]*	0.28 [0.07; 0.48]*
	Interaction (anxiety*sex)	–0.32 [–0.62, –0.01]*	–0.62 [–1.30; 0.07]	0.78 [0.22; 1.34]**	0.05 [–0.12; 0.22]	–0.06 [–0.19; 0.07]	0.04 [–0.09; 0.18]
Stress	Stress (centered), score	0.00 [–0.07; 0.08]	0.04 [–0.12; 0.20]	0.01 [–0.16; 0.17]	0.05 [0.00; 0.09]*	0.02 [–0.01; 0.06]	0.02 [–0.01; 0.06]
	Sex (women)[a]	–0.39 [–4.12; 3.34]	9.46 [3.81; 15.11]**	1.89 [0.32; 3.46]*	0.30 [–0.12; 0.71]	–0.18 [–0.57; 0.22]	0.18 [–0.23; 0.59]
	Time (12 weeks) [b]	–1.19 [–1.58; –0.81]***	–1.07 [–1.92; –0.21]*	3.90 [2.79; 5.01]***	–0.16 [–0.46; 0.15]	–0.29 [–0.51; –0.06]*	0.35 [0.16; 0.55]**
	Interaction (stress*sex)	–0.01 [–0.11; 0.09]	–0.02 [–0.25; 0.20]	–0.01 [–0.22; 0.20]	0.00 [–0.06; 0.05]	0.01 [–0.04; 0.05]	–0.03 [–0.07; 0.01]
Depression	Depression (centered), score	0.05 [–0.15; 0.24]	0.24 [–0.19; 0.66]	0.19 [–0.37; 0.74]	0.14 [0.00; 0.29]	0.05 [–0.06; 0.17]	0.07 [–0.03; 0.17]
	Sex (women)[a]	–0.44 [–4.13; 3.26]	9.37 [3.75; 15.00]**	1.80 [0.21; 3.39]*	0.25 [–0.17; 0.68]	–0.19 [–0.60; 0.22]	0.17 [–0.23; 0.57]
	Time (12 weeks)[b]	–1.12 [–1.52; –0.72]***	–1.01 [–1.89; –0.13]*	3.97 [2.83; 5.12]***	–0.10 [–0.41; 0.21]	–0.27 [–0.50; –0.04]*	0.34 [0.13; 0.55]**
	Interaction (depression*sex)	0.01 [–0.21; 0.23]	–0.27 [–0.75; 0.22]	–0.26 [–0.84; 0.33]	–0.05 [–0.21; 0.10]	0.00 [–0.12; 0.12]	–0.10 [–0.21; 0.01]
Well–being	Well–being (centered), score	0.01 [–0.03; 0.04]	0.01 [–0.06; 0.09]	–0.05 [–0.13; 0.03]	–0.01 [–0.03; 0.01]	0.00 [–0.02; 0.01]	–0.01 [–0.02; 0.01]
	Sex (women)[a]	–0.39 [–4.09; 3.31]	9.55 [3.92; 15.18]**	1.73 [0.20; 3.26]*	0.30 [–0.13; 0.72]	–0.18 [–0.58; 0.23]	0.19 [–0.23; 0.61]
	Time (12 weeks)[b]	–1.24 [–1.61; –0.89]***	–1.21 [–2.07; –0.34]**	4.11 [2.97; 5.25]***	–0.25 [–0.54; 0.05]	–0.34 [–0.55; –0.13]	0.35 [0.16; 0.55]**
	Interaction (well–being*sex)	–0.02 [–0.06; 0.02]	–0.01 [–0.10; 0.08]	0.06 [–0.03; 0.15]	–0.01 [–0.03; 0.02]	–0.01 [–0.03; 0.01]	0.01 [0.00; 0.03]

Flourishing	Flourishing (centered), score	0.01 [–0.09; 0.11]	–0.05 [–0.28; 0.17]	0.02 [–0.23; 0.26]	–0.08 [–0.14; –0.01]*	–0.03 [–0.08; 0.02]	–0.03 [–0.08; 0.01]
	Sex (women)[a]	–0.39 [–4.12; 3.34]	9.51 [3.87; 15.14]**	1.89 [0.33; 3.45]*	0.38 [–0.06; 0.82]	–0.13 [–0.56; 0.29]	0.21 [–0.20; 0.62]
	Time (12 weeks)[b]	–1.21 [–1.59; –0.83]***	–1.17 [–2.00; –0.33]**	3.87 [2.76; 4.98]***	–0.16 [–0.43; 0.11]	–0.30 [–0.51; –0.09]**	0.35 [0.16; 0.55]**
	Interaction (flourishing *sex)	–0.01 [–0.16; 0.14]	0.19 [–0.14; 0.52]	–0.01 [–0.30; 0.27]	0.02 [–0.05; 0.10]	0.00 [–0.07; 0.06]	0.03 [–0.03; 0.09]
Emotional eating	Emotional eating (centered), score	0.24 [–0.70; 1.18]	0.66 [–1.52; 2.83]	–1.05 [–3.27; 1.16]	-	0.63 [0.24; 1.02]**	0.21 [1.81; 2.52]***
	Sex (women)[a]	–0.50 [–4.19; 3.19]	9.39 [3.74; 15.04]**	2.12 [0.49; 3.75]*	-	–0.35 [–0.69; –0.02]*	0.16 [–0.26; 0.58]
	Time (12 weeks)[b]	–1.11 [–1.50; –0.73]***	–1.07 [–1.97; –0.18]*	3.73 [2.59; 4.87]***	-	–0.19 [–0.38; 0.00]*	0.35 [0.13; 0.56]**
	Interaction (emotional eating*sex)	0.18 [–0.86; 1.23]	–1.01 [–3.42; 1.40]	1.31 [–1.10;3.72]	-	–0.14 [–0.57; 0.29]	–0.25 [–0.75; 0.26]
Uncontrolled eating	Uncontrolled eating (centered), score	0.74 [0.00; 1.49]	1.06 [–0.69; 2.81]	–2.45 [–4.01; –0.89]**	0.35 [0.01; 0.69]*	-	–0.11 [–0.48; 0.25]
	Sex (women)[a]	–0.28 [–3.88; 3.31]	9.60 [4.03; 15.18]**	1.69 [0.33; 3.04]*	0.45 [0.17; 0.72]**	-	0.19 [–0.22; 0.59]
	Time (12 weeks)[b]	–0.93 [–1.33; –0.53]***	–0.98 [–1.92; –0.03]*	3.52 [2.44; 4.60]***	–0.04 [–0.29; 0.22]	-	0.27 [0.06; 0.49]*
	Interaction (uncontrolled eating*sex)	0.05 [–1.04; 1.14]	–1.21 [–3.74; 1.31]	3.10 [1.13; 5.07]**	0.73 [0.31; 1.16]**	-	–0.01 [–0.48; 0.47]
Cognitive restraint	Cognitive restraint (centered), score	–0.60 [–1.55; 0.35]	–1.10 [–3.24; 1.05]	2.63 [0.54; 4.71]*	0.10 [–0.54; 0.75]	–0.23 [–0.72; 0.26]	-
	Sex (women)[a]	–0.30 [–4.03; 3.42]	9.66 [4.02; 15.31]**	1.50 [–0.22; 3.21]	0.36 [–0.13; 0.85]	–0.10 [–0.54; 0.34]	-
	Time (12 weeks)[b]	–1.10 [–1.51; –0.69]***	–0.98 [–1.91; –0.06]*	3.42 [2.42; 4.42]***	–0.27 [–0.58; 0.04]	–0.30 [–0.53; –0.07]*	-
	Interaction (cognitive restraint*sex)	0.54 [–0.63; 1.72]	1.03 [–1.61; 3.66]	–2.13 [–4.53; 0.28]	–0.22 [–0.96; 0.52]	0.14 [–0.43; 0.71]	-

Values represent unstandardized regression coefficients (B). The specific "Predictor Variable" and "Interaction" terms in each column correspond to the variable named in the column header.
[a] The reference group for sex is men. [b] The reference group for time is baseline. * p<0.05; **p< 0.01, ***p< 0.001.
